# Heterogeneity of Collagen VI Microfibrils: STRUCTURAL ANALYSIS OF NON-COLLAGENOUS REGIONS*

**DOI:** 10.1074/jbc.M115.705160

**Published:** 2016-01-07

**Authors:** Tobias Maaß, Christopher P. Bayley, Matthias Mörgelin, Sandra Lettmann, Paolo Bonaldo, Mats Paulsson, Clair Baldock, Raimund Wagener

**Affiliations:** From the ‡ Center for Biochemistry, Medical Faculty,; ** Center for Molecular Medicine,; ‡‡ Cologne Excellence Cluster on Cellular Stress Responses in Aging-associated Diseases, and; §§ Center for Musculoskeletal Biomechanics, University of Cologne, D-50931 Cologne, Germany,; the § Wellcome Trust Centre for Cell-Matrix Research, University of Manchester, Manchester M13 9PT, United Kingdom,; the ¶ Department of Clinical Sciences, Division of Infection Medicine, Lund University, SE-221 84 Lund, Sweden, and; the ‖ Department of Molecular Medicine, University of Padova, 35131 Padova, Italy

**Keywords:** collagen, electron microscopy (EM), extracellular matrix, single particle analysis, small-angle x-ray scattering (SAXS), VWA domain, microfibrils

## Abstract

Collagen VI, a collagen with uncharacteristically large N- and C-terminal non-collagenous regions, forms a distinct microfibrillar network in most connective tissues. It was long considered to consist of three genetically distinct α chains (α1, α2, and α3). Intracellularly, heterotrimeric molecules associate to form dimers and tetramers, which are then secreted and assembled to microfibrils. The identification of three novel long collagen VI α chains, α4, α5, and α6, led to the question if and how these may substitute for the long α3 chain in collagen VI assembly. Here, we studied structural features of the novel long chains and analyzed the assembly of these into tetramers and microfibrils. N- and C-terminal globular regions of collagen VI were recombinantly expressed and studied by small angle x-ray scattering (SAXS). *Ab initio* models of the N-terminal globular regions of the α4, α5, and α6 chains showed a C-shaped structure similar to that found for the α3 chain. Single particle EM nanostructure of the N-terminal globular region of the α4 chain confirmed the C-shaped structure revealed by SAXS. Immuno-EM of collagen VI extracted from tissue revealed that like the α3 chain the novel long chains assemble to homotetramers that are incorporated into mixed microfibrils. Moreover, SAXS models of the C-terminal globular regions of the α1, α2, α4, and α6 chains were generated. Interestingly, the α1, α2, and α4 C-terminal globular regions dimerize. These self-interactions may play a role in tetramer formation.

## Introduction

Collagen VI is a widely expressed member of the triple helix-containing protein superfamily of collagens (1). In contrast to the abundant fibril-forming collagens I and II, collagen VI forms beaded microfibrils that anchor large interstitial structures (2). Other extracellular matrix proteins bind to the non-collagenous domains and are important for the embedding of these microfibrils in the matrix (3–6). Collagen VI has the lowest triple helix content among all collagens and is predominantly composed of globular VWA^4^ domains (7).

**FIGURE 1.**
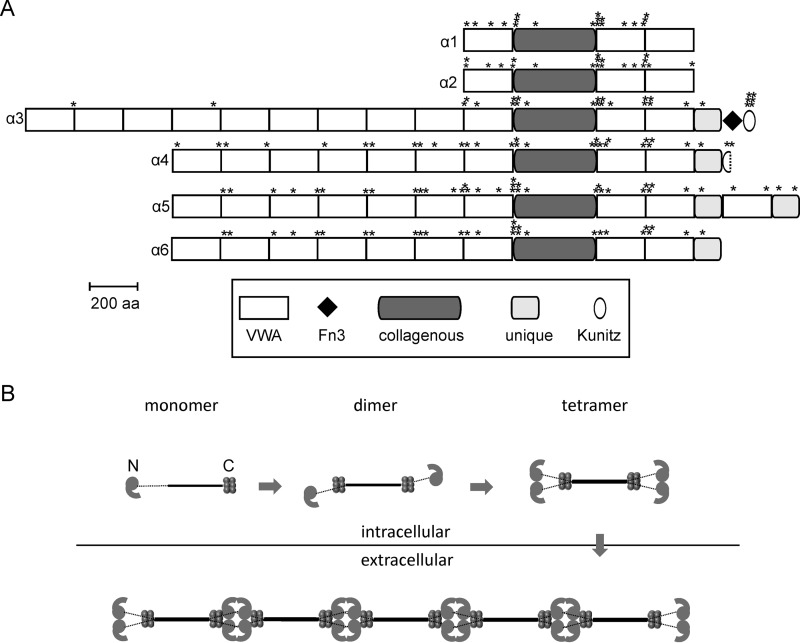
**Domain structure (*A*) and assembly (*B*) of collagen VI chains.** *A,* positions of the cysteine residues are marked by *asterisks* above each chain. The domain structure is as shown in Ref. 50. *VWA*, von Willebrand factor type A domain, Fn3, Fibronectin type 3 domain. *B,* intracellularly the heterotrimeric monomers assemble to tetramers that are secreted and form microfibrils in the extracellular space. Adapted from Ref. 24.

For many years collagen VI was thought to consist of only the shorter α1 and α2 chains and the longer α3 chain. Intracellularly, heterotrimeric monomers are formed that assemble to dimers in an antiparallel fashion. These subsequently assemble laterally to tetramers, are secreted, and finally associate end to end to form beaded microfibrils (Fig. 1) (8, 9). More recently, three novel long chains, α4, α5, and α6, were identified (Fig. 1) that are thought to substitute for the α3 chain in the assembly (10, 11). Interestingly, humans and modern apes lost the α4 chain by a large pericentral inversion on chromosome 3 (12). The novel long chains have a restricted, often complementary expression at certain basement membranes (13).

Mutations in the α1, α2, and α3 chains cause collagen VI-related myopathies (14) ranging from the severe Ullrich congenital muscular dystrophy to the milder Bethlem myopathy, including intermediate forms (15). Mutations in the α1 and α2 chains can also lead to limb-girdle muscular dystrophy (16) and a mutation in the α2 chain to myosclerosis (17). Recently, early onset isolated dystonia, a neurological disease, was shown to be caused by mutations in the α3 chain (18). Interestingly, a possibly digenic myopathy patient carried compound heterozygous mutations in the α6 chain and a mutation in the α3 chain (19).

Collagen VI α1 knock-out mice that develop a mild form of myopathy (20) were used for in depth studies of the myopathy pathomechanism. The muscles of the knock-out mice lose their contractile strength and display a marked dilation of the sarcoplasmic reticulum. Abnormal mitochondria are seen in muscle cells, with altered cristae and dense bodies and an opening of the mitochondrial permeability transition pore leading to enhanced apoptosis (21). The persistence of abnormal mitochondria and apoptosis is caused by a disturbed autophagy (22). Moreover, the mice show impaired muscle regeneration and reduced satellite cell self-renewal after injury (23). However, little is known about the structural consequences of patient mutations that do not affect the formation of the triple helix.

The structure of the non-collagenous parts of collagen VI has mainly been studied by use of electron microscopy. Moreover, by SAXS it was shown that the extended array (N9 to N1) of N-terminal VWA domains of the α3 chain has a tight C-shape. Indeed, in collagen VI bead regions, the N-terminal globules predominantly consisting of α3 chain VWA domains project out from the globular bead region like angled radial spokes. These could potentially provide interaction surfaces for other cell surface or matrix molecules (24). Only two collagen VI domain structures have been resolved to high resolution, the Kunitz domain (25) and the N5 VWA domain of the α3 chain (26). The latter structure showed that in contrast to VWA domains in other proteins, a C-terminal extension is present that places the N and C termini at opposite sides of the globular domains. This allows a near-linear arrangement of the adjacent VWA domains and is in good agreement with the SAXS model of the N9 to N1 fragment of the α3 chain. As a consequence of the sparse structural information, only few aspects of the complex assembly are understood at atomic resolution. It was proposed that alternating hydrophobic and ionic interactions between the antiparallel triple helices (27) and interactions between the triple helix and the C2 domain of the α2 chain are important for dimer formation (28). Moreover, it was shown that at least the N5 to N1 domains of the α3 chain are important for the microfibril formation (29) and that the Kunitz domain can be cleaved off immediately after fibril formation (30). Studies on the impact of mutations found in myopathy patients also gave insight into the role of different domains, *e.g.* the critical importance of a correctly folded C1 domain of the collagen VI α2 chain in microfibril formation (31).

Nevertheless, most aspects of structure and assembly of collagen VI and especially of the contribution of the novel long chains are not known. Which are the spatial structures of their non-triple helical parts? Do they assemble into tetramers, as has been shown for collagen VI molecules containing the α3 chain? How are they involved in fibril formation? We therefore performed a comprehensive study to determine structures within the novel long chains and to analyze the composition and assembly of α4, α5, and α6 chains containing collagen VI tetramers and microfibrils.

## Experimental Procedures

### 

#### 

##### Recombinant Expression and Purification of N- and C-terminal Domains of Collagen VI α Chains and Generation of Specific Antibodies

The cDNA constructs coding for the non-collagenous domains of collagen VI were generated by RT-PCR on total RNA from brain or intestine and cloned with 5′-terminal NheI or XhoI and 3′-terminal BamHI or XhoI restriction sites (Table 1). Each of the amplified PCR products were inserted into a modified pCEP-Pu vector containing an N-terminal BM-40 signal peptide and a C-terminal One-STrEP tag downstream of the restriction sites (32). HEK293-EBNA cells (Invitrogen) were transfected with the recombinant plasmids using the FuGENE 6 reagent (Roche Applied Science, Mannheim, Germany) according to the manufacturer's protocol. The cells were selected with puromycin (1 μg/ml), and the recombinant proteins were purified directly from serum-containing cell culture medium. After filtration and centrifugation (1 h, 10,000 × *g*), the cell culture supernatants were applied to a StrepTactin column (1.5 ml, IBA GmbH) and eluted with 2.5 mm desthiobiotin, 10 mm Tris-HCl, pH 8.0. The purified recombinant C-terminal collagen VI fragments were used to immunize rabbits. To abolish cross-reactivity, the antisera were depleted on columns coupled with all other N- and C-terminal fragments and specific antibodies purified by affinity chromatography on a column with antigen coupled to CNBr-activated Sepharose (GE Healthcare). The specific antibodies were eluted with 0.1 m glycine, pH 2.5, and the eluate was neutralized with 1 m Tris-HCl, pH 8.8, and adjusted to 150 mm NaCl.

**TABLE 1:** **Primers for expression constructs** The following abbreviations are used: fw, forward; rev, reverse. The restriction enzyme recognition sites are underlined.

Chain	5′–3′Sequence	Restriction enzyme
α3N	tatctcgagctgatggatctgctgtgaggtta (fw)	XhoI
	aggaaccagggatcccaggggcctgtcatacatgaagcc (rev)	BamHI
α4N	attgctagcccacagaggattgtctgcagggaggcgtctg (fw)	NheI
	aggctcgagcagtaagacgcttgggagg (rev)	XhoI
α5N	attgctagcttagaccagagcccagggccaggccccgagta (fw)	NheI
	atactcgaggagcgtcttgcccaggtctagcatcccaatgg (rev)	XhoI
α6N	attgctagccaagattctggccccgagtacgcagac (fw)	NheI
	tatctcgagcagctgactcccaaggttcc (rev)	XhoI
α1C	gcagctagctgcacatgtggacccattga (fw)	NheI
	aacctcgaggcccagtgccaccttcct (rev)	XhoI
α2C	gaagctagctgtgagaagcgctgtggt (fw)	NheI
	gcaggatccacagatccagcggatg (rev)	BamHI
α3C	aaagctagcctggagtgccctgtattcccaac (fw)	NheI
	tttggatcctcaaactgttaactcaggactac (rev)	BamHI
α4C	attgctagcggtatttccaggtgcccag (fw)	NheI
	agcggatcccttcaagacataattcaggc (rev)	BamHI
α5C	attgctagcgacaagtgccctgtgtatcc (fw)	NheI
	agcggatcctcttgcatcttcgccatcttg (rev)	BamHI
α6C	attgctagcggaaaacctgagtgcccagt (fw)	NheI
	agcggatccggcacttcgtttaccgagttt (rev)	BamHI

##### Gel Electrophoresis and Immunoblot

Collagen VI was purified from whole newborn mouse carcasses as described (33). Samples were subjected to SDS-PAGE on 8% (w/v) polyacrylamide gels under reducing conditions (5% β-mercaptoethanol). Proteins were electrophoretically transferred to Immobilon-P membranes (Millipore). The collagen VI chains were detected with affinity-purified antibodies raised against recombinant C-terminal regions. Secondary antibodies conjugated with horseradish peroxidase were used for detection by chemiluminescence (ECL). Collagen VI purified from whole newborn mouse carcasses and treated with 2 m urea was submitted to electrophoresis on 0.5% agarose, 2.4% polyacrylamide composite gels under non-reducing conditions (34). IRDye700DX-conjugated sheep anti-rabbit IgG and IRDye800CW-conjugated goat anti-guinea pig IgG (Rockland) secondary antibodies were applied for multiplex detection, and bands were visualized using the Odyssey Infrared Imaging System.

##### SAXS Data Collection and Processing

All protein samples were purified by size exclusion chromatography using a Superdex200 column on an ÄKTA Purifier FPLC in 10 mm phosphate buffer, 400 mm NaCl, pH 7.4, and concentrated using Vivaspin centrifugal concentrators if required. SAXS measurements of the N-terminal α4, α5, and α6 collagen VI chains (∼6 mg/ml) with matched buffer blanks were collected on the I22 beamline at Diamond Light Source. The covered range of momentum transfer was 0.014 < *q* < 0.48 Å^−1^ (*q* = 4 πsinθ/λ) with detector distance of 2.5 m and x-ray wavelength of 0.1 nm. Data were collected in 120 successive 1-s frames to check for radiation damage. The data were normalized to the intensity of the incident beam and spherically averaged using an in-house program. Initial data processing and buffer subtraction were done in PRIMUS (35). SAXS measurements of the C-terminal collagen VI chains (α1, 4.3 mg/ml; α2, 4 mg/ml; α4, 3.5 mg/ml; α6, 3.5 mg/ml) with matched buffer blanks were collected at the EMBL-P12 beamline at PETRAIII (DESY, Hamburg, Germany) employing automated data acquisition and radial averaging protocols (36). For all data, the forward scattering intensity, *R_g_*, and distance distribution function *p*(*r*) were evaluated with GNOM (37), and particle shapes were restored *ab initio* using DAMMIN (38).

Multiple runs were performed to generate 20 models that were combined and filtered to produce an averaged model using the DAMAVER (39) software package. χ fitting values and the normalized spatial discrepancy for *ab initio* modeling is shown in Table 2. Rigid body modeling against the experimental SAXS data was performed with SASREF (40) using individual VWA domains with a distance range of 10–15 Å between each domain. Because the N-terminal regions were flexible, they were analyzed as an ensemble; 10,000 models were generated using RANCH, and an ensemble of models representing the experimental SAXS data were generated by EOM (41).

**TABLE 2:** **SAXS values** NSD is normalized spatial discrepancy. ND means not determined.

Construct	DAMMIN χ range	Damsel NSD	SASREF χ range
α1C	1.366–1.380	0.88	1.16–1.21
α2C	0.879–0.887	0.80	0.74–0.97
α4N	0.718–0.793	0.74	1.09–1.10
α4C	1.025–1.028	0.69	ND
α5N	1.063–1.089	0.78	ND
α6N	0.604–0.610	0.81	ND
α6C	1.187–1.196	0.66	ND

##### Electron Microscopy and Single-particle Analysis

Collagen VI α4, α5, and α6 N-terminal regions (∼50 μg/ml) were adsorbed onto glow-discharged carbon-coated grids and stained with 4% (w/v) uranyl acetate, pH 4.7. Grids were observed using an FEI Tecnai Twin (120 keV) transmission EM, and images were recorded under low dose conditions (<10 e^−^/Å^2^) on 2048 × 2048 pixel CCD camera at ×30,000 (2.8 Å/pixel) between −0.2- and −2.0-μm defocus. Imagic-5 (42) was used for particle picking and image processing. Images were contrast transfer function corrected, and the total numbers of particles in each dataset were 6047 for α4; 5552 for α3; 2745 for α5, and 2129 for α6. Characteristic class-sum images were used as references to align the dataset. For the α4 chain, angular reconstitution produced unique projection classes, enabling calculation of an initial three-dimensional reconstruction that was then subjected to five rounds of iterative refinement. The resolution was estimated by Fourier shell correlation as ∼40 Å using the 0.5 correlation criteria.

##### Immunoelectron Microscopy on Natively Extracted Microfibrils

Native collagen VI microfibrils were extracted from E14.5 mouse lungs by collagenase digestion in analogy to previously described protocols for bovine cornea (43) with modifications as described in Ref. 44. The distribution of the different α chains in collagen VI preparations was analyzed by incubation for 30 min at 37 °C with colloidal gold-labeled (45) antibodies specific for the N-terminal regions of the long collagen VI α chains (13) followed by negative staining and transmission electron microscopy as described (46). Specimens were observed in a Philips/FEI CM 100 TWIN transmission electron microscope (FEI Co.) at 60-kV accelerating voltage. Images were recorded with a side-mounted Olympus Veleta camera with a resolution of 2048 × 2048 pixels using ITEM^TM^ software. All electron microscopic work was performed at the Core Facility for Integrated Microscopy, Panum Institute, University of Copenhagen.

## Results

### 

#### 

##### Recombinant Expression of N- and C-terminal Globular Regions of Collagen VI

**FIGURE 2.**
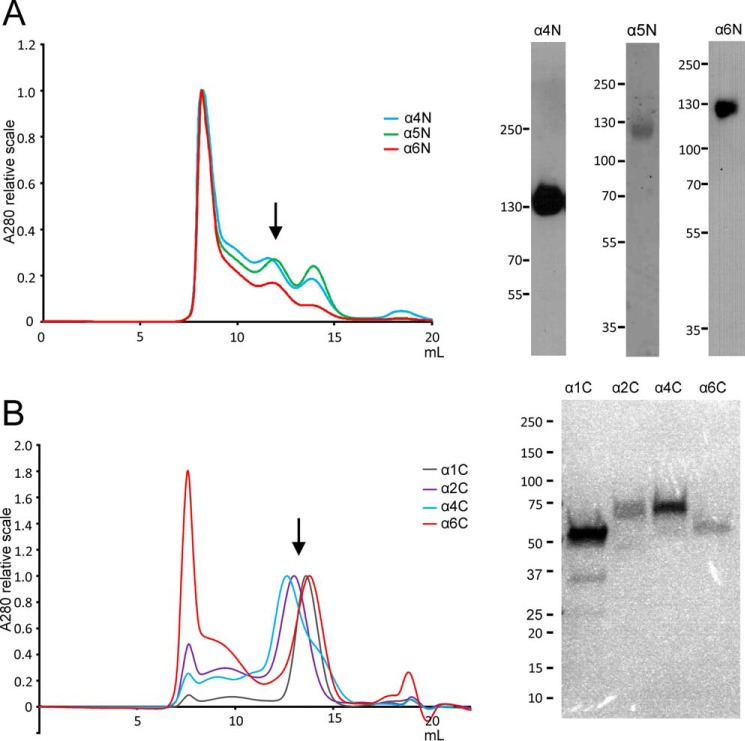
**Purification of recombinant N-terminal (*A*) and C-terminal (*B*) regions of collagen VI α1, α2, α4, α5, and α6 chains.** *Left side,* size-exclusion chromatography (example runs are shown) was performed to further purify the protein samples that were obtained after affinity purification and to remove higher aggregates. Absorption was normalized for the main peaks of the different chromatograms. Collected fractions are indicated by *arrows. Right side,* purity of the fractions was evaluated by SDS-PAGE and subsequent Ponceau staining (α5 chain) (*A*) or Western blotting (α4 and α6 chain) using chain-specific antibodies (*A*) or Coomassie staining (*B*).

The globular non-collagenous regions of the different collagen VI α chains are important for assembly and structure of the complex microfibrillar network that is present in nearly all tissues. Therefore, N- and C-terminal globular regions of the α1–α6 chains of collagen VI were recombinantly expressed in the eukaryotic HEK293-EBNA cells and used for structural analysis. The proteins were tagged with the One-STrEP-tag and affinity-purified. SDS-PAGE often revealed a heterogeneous band pattern, both under reducing and non-reducing conditions due to proteolytic processing and the formation of disulfide-linked oligomers (data not shown). For some experiments, the proteins were therefore further purified by size-exclusion chromatography (Fig. 2). Some of the recombinant proteins (α3C–α6C) were used for immunization of rabbits and guinea pigs to generate antisera specific for the respective globular regions.

##### Ab Initio SAXS Models of the N-terminal Globular Regions of the α4, α5, and α6 Chains Indicate a C-shaped Structure

**FIGURE 3.**
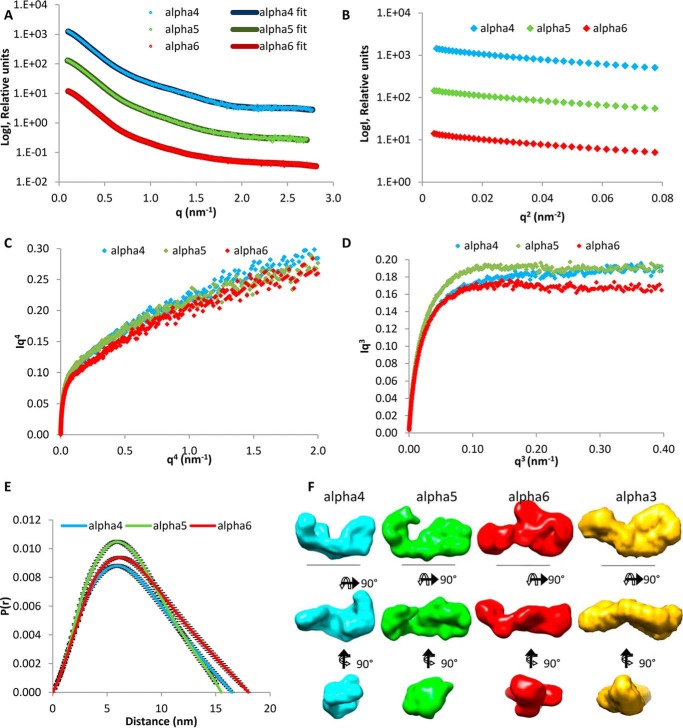
**SAXS data collected for the N-terminal regions of the collagen VI α4, α5, and α6 chains.** *A,* SAXS profile showing the log of x-ray scattering intensity (*LogI*) as a function of the scattering vector *q* for the experimental scattering data with the DAMMIN fit with lowest χ value superimposed. *B,* Guinier plot (logI *versus q*^2^) of the low *q* region of the x-ray scattering data where the radius of gyration (*R_g_*) can be measured from the gradient of the slope (−*R_g_*^2^/3). *C, q*^4^ plot does not show a plateau, whereas the *q*^3^ plot (*D*) has a linear plateau that indicates some flexibility in the N-terminal regions. *E, P*(*r*) distribution plot shows the probability of different distances found within the protein. The longest distance is indicated by the *D*_max_. *F, ab initio* models generated from the SAXS data shown in three orthogonal orientations. *Scale bar,* 10 nm. For all panels, the α4, α5, and α6 chains are shown in *blue, green,* and *red*, respectively. The *ab initio* model of the α3 N9 to N1 region from Ref. 24 is shown in *yellow* for comparison.

The first step was to determine the structure of the large N-terminal regions of the novel α4, α5, and α6 chains that resemble the α3 chain by sequence and domain structure and are thought to replace the α3 chain in some collagen VI molecules (11). It was therefore relevant to determine the shape of the N-terminal globular regions for these chains that could then be compared with a SAXS model of the large N-terminal globular region of the collagen VI α3 chain. It has been shown that this region has a compact C-shaped structure that is likely to participate in the assembly of collagen VI (24). SAXS measurements were performed at the Diamond Light Source, beamline I22. Size-exclusion chromatography was carried out upstream of the beamline and the monomer fractions used (Fig. 3*A*). Guinier plots of the x-ray scattering results allowed assessment of data quality (Fig. 3*B*). In contrast to the *q*^4^ plot (Fig. 3*C*), the *q*^3^ plot (Fig. 3*D*) showed a linear plateau that indicates some flexibility in the N-terminal regions (47). Distance distribution plots revealed longest distances of 15.6, 16.5, and 18.0 nm for the α5, α4, and α6 chains, respectively (Fig. 3*E*), and *ab initio* models were generated (Fig. 3*F*) with DAMMIN (38). All three models display a C-shaped conformation reminiscent of that of the α3 chain N-terminal globular region (Fig. 3*F*) (24).

##### Single Particle EM Nanostructure of the N-terminal Globular Region of the α4 Chain Confirms the C-shaped Structure Revealed by SAXS

**FIGURE 4.**
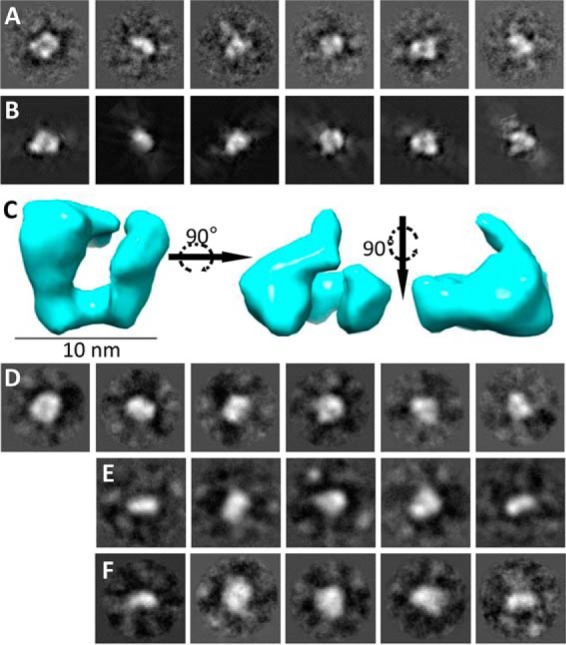
**Nanostructure of the N-terminal region of the collagen VI α4 chain and comparison with the N-terminal regions of the α3, α5, and α6 chains.** *A,* representative class averages of the α4 chain generated by reference-free classification using the IMAGIC-5 image processing and analysis software (Image Science, Germany). These class averages were used to generate the three-dimensional reconstruction. *B,* back-projections of the three-dimensional reconstruction, showing similarity to corresponding class averages. *C,* three-dimensional reconstruction of the N-terminal region of the α4 chain generated by single particle analysis shown in three orthogonal views. *Scale bar,* 10 nm. Representative class averages generated by reference-free classification using the IMAGIC-5 software for the α3 (*D*), α5 (*E*), and α6 chains (*F*). All *boxes* have a size of 35.8 × 35.8 nm.

**FIGURE 5.**
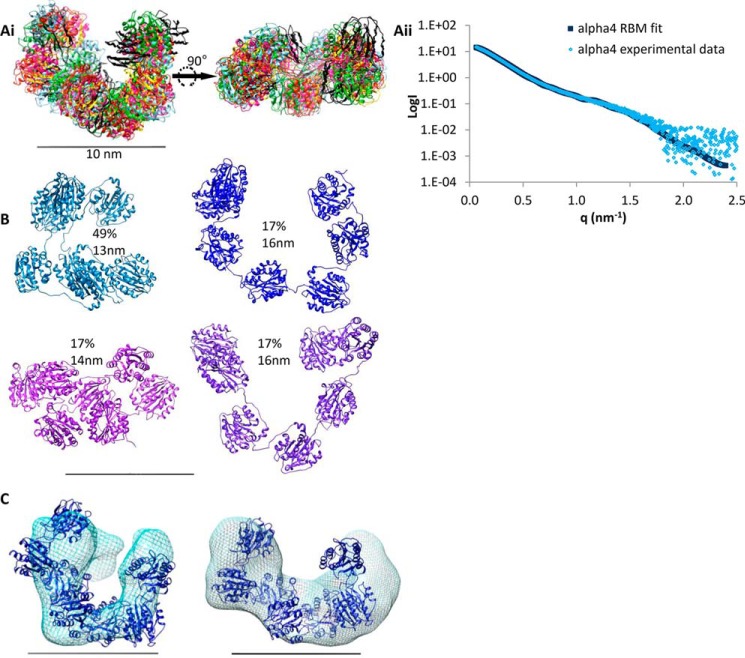
**Rigid body modeling of the N-terminal region of the collagen VI α4 chain and comparison with SAXS *ab initio* and EM data.** *A, panel i,* six superimposed RBM simulations for the N-terminal region of the α4 chain shown in two orientations and the theoretical scattering of the rigid body modeling with lowest χ value compared with the experimental scattering data (*A, panel ii*). *B,* ensemble of models produced using EOM from the x-ray scattering data and their relative proportions and maximum dimension (*D*_max_). *C,* EM map (*left*) and *ab initio* model (*right*) for the N-terminal region of the α4 chain both with a representative model superimposed. For all panels the *scale bar* is 10 nm.

To confirm the SAXS models, single particle EM was performed after negative staining with uranyl acetate. The N-terminal globular region of the α4 chain was chosen as a prototype. 6047 single molecules were selected with the Imagic-5 program (42), and reference-free class averages were generated (Fig. 4*A*) and used for the calculation of a three-dimensional model (Fig. 4*C*). A structure similar to the C-shaped SAXS model was obtained (Fig. 3*F*). The quality of the model was tested by back-projections showing similarity to the corresponding class averages (Fig. 4*B*). Single particle EM was also performed on the N-terminal globular regions of the α3, α5, and α6 chains (Fig. 4, *D–F*). The general appearance of the class averages was similar, but the numbers of examined single molecules and orientations observed were not sufficient to obtain a three-dimensional structure. Recently, the crystal structure of a single collagen VI VWA domain (α3N5) was determined (26). This structure was used for rigid body modeling of the α4 chain N terminus to model domain organization from the SAXS data. The program SASREF (40) was used and generated six models that were similar and could be superimposed to show a C-shaped structure (Fig. 5*A*). As the *q*^4^ plot suggested flexibility in the N-terminal regions (Fig. 3*C*), ensemble analysis was also used to model different conformers. 10,000 models were generated of the α4 chain N terminus and compared with the experimental scattering data using EOM (41). An ensemble of four models was predicted with an average *D*_max_ of 14.8 nm (Fig. 5*B*). The ensemble included two models that contributed 34% of the ensemble with an open C-shape (*D*_max_ = 16.4 nm), and one model contributing 49% of the ensemble had a more compact C-shape (*D*_max_ = 13.3 nm). The more open shapes have a similar *D*_max_ to that predicted from the experimental data (16.5 nm). Comparing the *ab initio* SAXS model and the EM-based model revealed the strongest agreement with the rigid body model that had an intermediate conformation between the open and closed C-shape. The convergence of different low resolution structural data on a C-shaped model reflects the reliability of this conformation of the N-terminal globular region of the α4 chain (Fig. 5*C*).

##### Collagen VI Extracted from Tissue Contains Novel Long Chains Incorporated into Mixed Microfibrils

**FIGURE 6.**
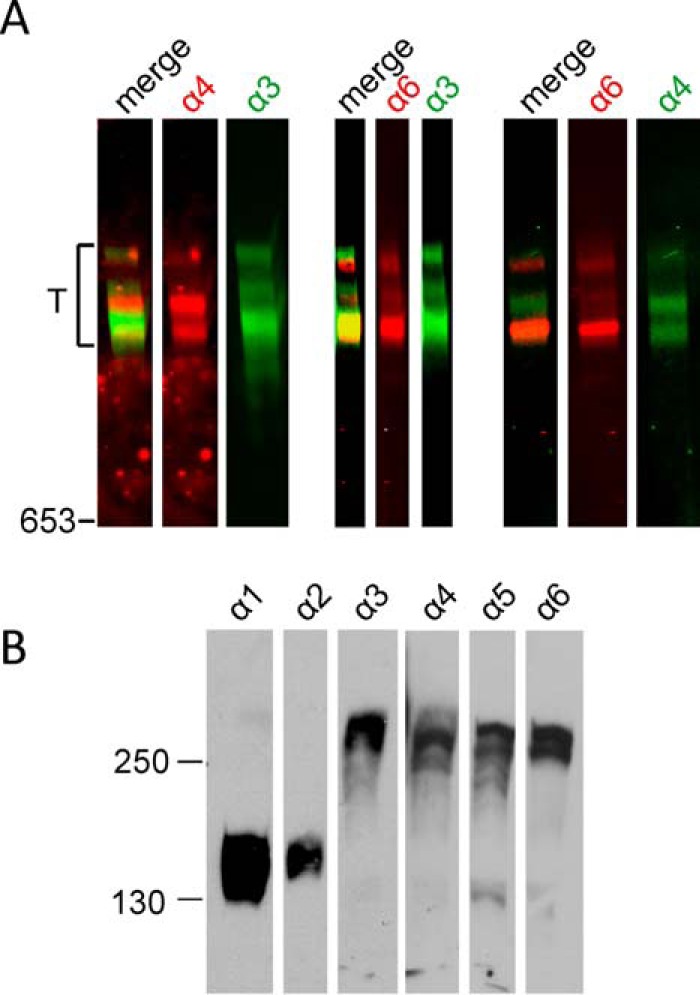
**Immunoblot analysis of collagen VI.** *A,* collagen VI purified from whole newborn mouse carcasses was separated in agarose/polyacrylamide composite gels under non-reducing conditions and was detected with affinity-purified chain-specific long chain antibodies from rabbit (α4 and α6, *red*) and guinea pig (α3 and α4, *green*) and secondary antibodies labeled with spectrally distinct infrared fluorescent dyes. The relative mobility of tetramers is indicated (*T*). *B,* collagen VI purified from whole newborn mouse carcasses was separated in normal SDS-polyacrylamide gels under reducing conditions and detected with polyclonal antibodies specific for the collagen VI α1–α6 chains.

**FIGURE 7.**
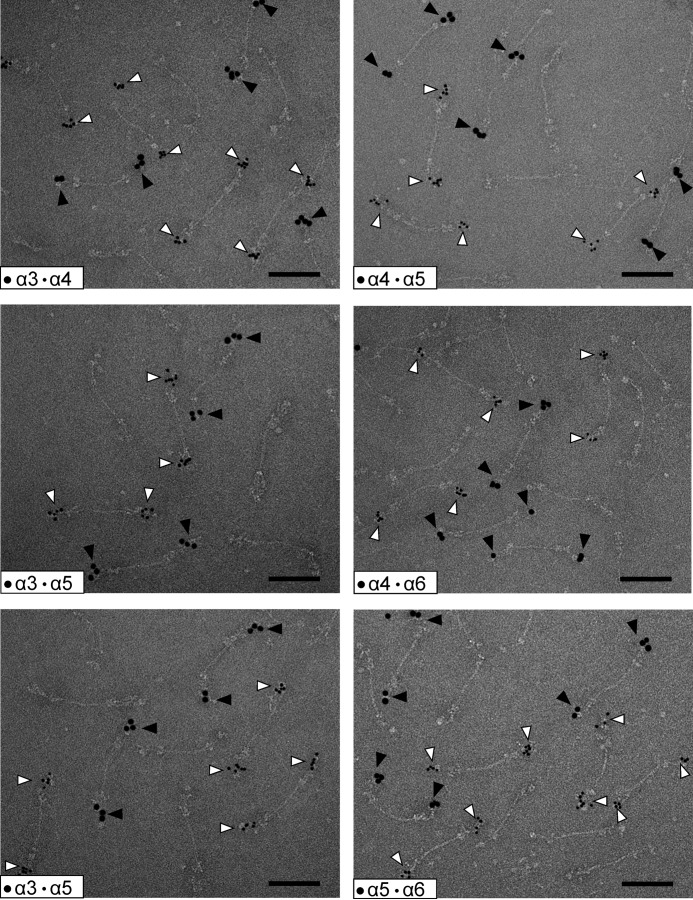
**Composition of collagen VI tetramers as detected by electron microscopy after negative staining.** Representative collagen VI tetramers from E14.5 mouse lung containing different long α chains are shown. Only homotetramers were detected. The N-terminal regions of the long chains were doubly labeled using specific gold-labeled antibodies against the different α chains. Small gold particles (*open arrowheads*) always stain the α chain with the lower number, whereas the large gold particles (*filled arrowheads*) stain that with the higher number. *Scale bar,* 100 nm.

**FIGURE 8.**
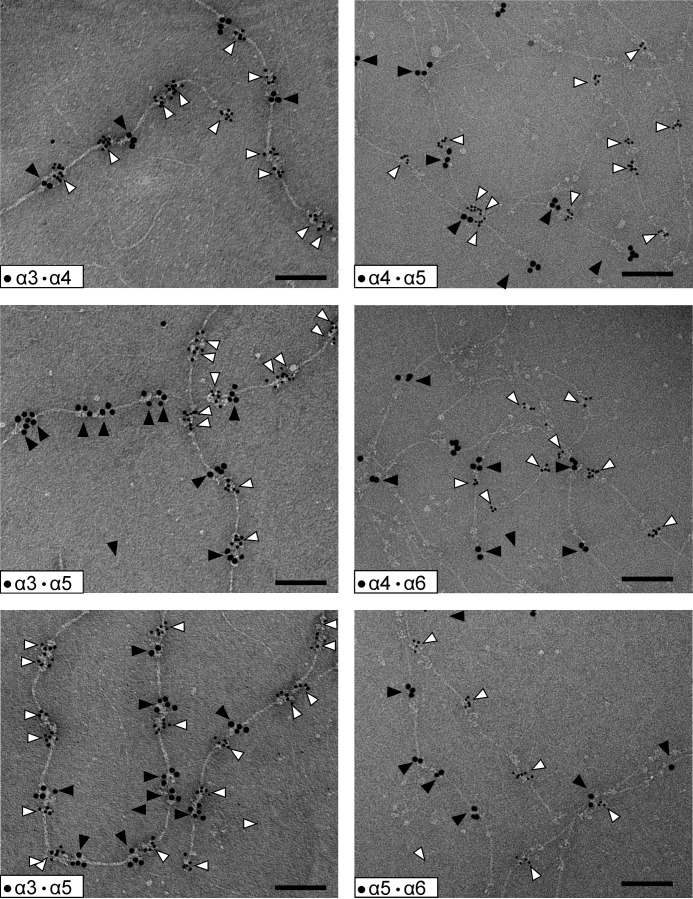
**Structure of collagen VI microfibrils as detected by electron microscopy after negative staining.** Representative collagen VI microfibrils from E14.5 mouse lung composed of tetramers containing different long chains are shown. Only homotetramers were detected. The N-terminal regions of the long chains were double labeled using specific gold-labeled antibodies against the different α chains. Small gold particles (*open arrowheads*) always stain the α chain with the lower number, whereas the large gold particles (*filled arrowheads*) stain that with the higher number. Note that as the tetramers overlap the “outer” globules belong to the same tetramer, and the “inner” globules belong to the neighboring tetramers. This is indicated in Fig. 11 in a schematic drawing of the mixed α3/α6 microfibril. *Scale bar,* 100 nm.

Because we showed that the N-terminal regions of the novel long collagen VI chains, α4, α5, and α6, have a similar structure as the α3 chain, the next question was whether they form tetramers and microfibrils similar to those that contain the α3 chain? So far, their assembly into higher order structures has been only sparsely studied. However, the novel long chains are lacking in the extracellular matrix of Col6a1^−/−^ mice, indicating that they are contained in or associated with the classical collagen VI microfibrils (13). Three important questions remain. 1) Do the novel long collagen VI chains also assemble into tetramers, as has been shown for collagen VI molecules containing the α3 chain? 2) If so, what is the composition of the tetramers, are they homotetramers^5^ or heterotetramers with regard to long chain composition? 3) Do collagen VI molecules containing the novel long chains assemble into microfibrils at all and, if so, what is the composition of these? To answer these questions, we studied collagen VI extracted from tissue. First, we used collagen VI purified from whole newborn mouse carcasses. By electrophoresis on composite SDS agarose/polyacrylamide gels we could show that collagen VI oligomers containing the α4 or α6 chains migrate to positions that are similar to those of tetramers containing the α3 chain (Fig. 6*A*). By multiplex detection, using affinity-purified chain-specific long chain antibodies from rabbit and guinea pig and secondary antibodies labeled with spectrally distinct infrared fluorescent dyes, we could detect bands that migrate to both similar and different positions. The bands that migrate at similar positions could either be heterotetramers or homo-oligomers that coincidently co-migrate due to similar size. However, there are bands that migrate at positions where mostly no other bands appear, *e.g.* the major α4 chain band. This clearly indicates that at least a portion of the long chains form homo-oligomers. The α5 chain could only be detected by SDS-PAGE under reducing conditions (Fig. 6*B*), and for this chain it was therefore not possible to determine whether it forms homo-oligomers or heterotetramers. Neither could the composition of the co-migrating α4 or α6 chain tetramers be determined. Only a method where individual microfibrils can be studied by use of chain-specific antibodies is suitable to address this question. Consequently, immunoelectron microscopy was performed on natively extracted collagen VI from E14.5 mouse lung where all four long chains are expressed. Interestingly, we could only detect homotetramers, irrespective of whether single tetramers (Fig. 7) or microfibrils were labeled (Fig. 8). In microfibrils, the “outer” globules representing the N-terminal globules were always labeled with the same antibody. As the assembled tetramers overlap, these belong to the same tetramer, in contrast to the “inner” globules that belong to the neighboring tetramers. Tetramers stained with two different antibodies were not observed. Strikingly, in all double labelings using all combinations of antibodies, we could identify mixed microfibrils composed of homotetramers containing different long α chains throughout.

##### Models of the C-terminal Globular Regions of the α1, α2, α4, and α6 Chains Give Insight into Their Role during Assembly

**FIGURE 9.**
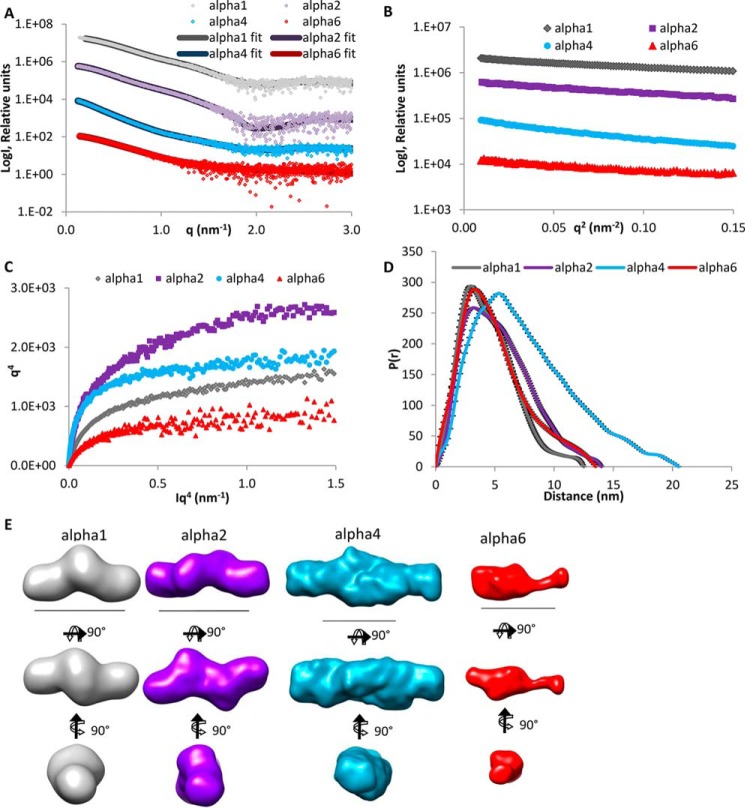
**SAXS data collected for the C-terminal regions of the collagen VI α1, α2, α4, and α6 chains.** *A,* SAXS profile showing the log of x-ray scattering intensity (*LogI*) as a function of the scattering vector *q* for the experimental scattering data with the DAMMIN fit with lowest χ value superimposed. *B,* Guinier plot (logI *versus q*^2^) of the low *q* region of the x-ray scattering data where the radius of gyration (*R_g_*) can be measured from the gradient of the slope (−*R_g_*^2^/3). *C, q*^4^ plot has a linear plateau that indicates that these regions are not flexible. *D, P*(*r*) distribution plot shows the probability of different distances found within the protein. The longest distance is indicated by the *D*_max_. *E, ab initio* models generated from the SAXS data shown in three orthogonal orientations. *Scale bar,* 10 nm. For all panels, the α1, α2, α4, and α6 chains are shown in *gray, purple, blue,* and *red*, respectively.

**FIGURE 10.**
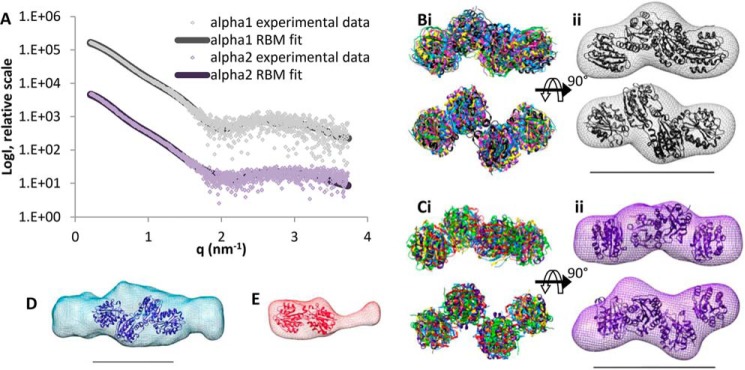
**Rigid body modeling of collagen VI C-terminal regions and comparison with SAXS *ab initio* models.** *A*, theoretical scattering of representative RBMs was compared with the experimental scattering data of the C-terminal regions of the α1 and α2 chains. *B, panel i,* six superimposed RBM simulations for the C-terminal region of the α1 chain, and *panel ii, ab initio* model for the C-terminal region of the α1 chain with a representative RBM superimposed, both shown in two orientations. *C,* similar to the C-terminal region of the α2 chain. *D,* two VWA domain pairs superimposed into the α4 chain *ab initio* model indicating that the C-terminal region of the α4 chain is dimeric with four VWA domains accommodated in the central density and two symmetric protrusions. *E,* however, the C-terminal region of the α6 chain is monomeric and can only accommodate one VWA domain pair and the additional domain is seen as an elongated protrusion. For all panels the *scale bar* is 10 nm.

Having shown that the shapes of the N-terminal domains of long collagen VI α chains are similar and that these chains therefore have the potential to replace each other in assembly, we wanted to provide structural information on the C-terminal regions of the different α chains, as at least in the α1 and α2 chains these regions appear to be involved in collagen VI assembly (48). So far, except for the Kunitz domain (25) of the α3 chain, there is no structural information on the C-terminal globular regions of the collagen VI chains beyond single EM images. We therefore performed SAXS measurements on the C-terminal fragments of the α1, α2, α4, and α6 chains at PetraIII (beamline P12) (Fig. 9*A*). The C-terminal fragments of the α3 and the α5 chains could not be purified at the concentrations that are required for SAXS measurements. Guinier plots of the x-ray scattering data allowed assessment of data quality (Fig. 9*B*). The *q*^4^ plots showed a linear region indicating little flexibility in the C-terminal regions (Fig. 9*C*). Distance distribution plots revealed longest distances of 12.5, 14.1, 20.5, and 13.4 nm for the α1C, α6C, α4C, and α2C chains, respectively (Fig. 9*D*). Interestingly, the molecular weight of the C-terminal globular regions of the α1, α2, and α4 chains is consistent with dimers from the volume of correlation (49), and these were processed with P2 symmetry. *Ab initio* modeling revealed elongated structures for each α chain (Fig. 9*E*). Because α1C and α2C, like the N-terminal globular regions, consist only of VWA domains, rigid body modeling using the structural data from the N5 domain of the α3 chain was performed (Fig. 10*A*). The models fit well into the *ab initio* SAXS model, and the α1C and α2C structures are very similar. In each case, dimerization occurs via a contact between one of the tandem VWA domains leading to a staggered “zigzag” conformation (Fig. 10, *B* and *C*). α4C and α6C contain other domains without known structure in addition to VWA domains, and therefore rigid body modeling could not be performed. However, four VWA domains can be accommodated in the central density of the *ab initio* model of α4C, and one pair of VWA domains in the *ab initio* model of α6C where the additional unique domain is seen as an elongated protrusion (Fig. 10, *D* and *E*).

## Discussion

The formation of collagen VI microfibrils is a complex multistep process. Already in the secretory pathway 12 α chains assemble to tetramers that have a molecular mass similar to that of ribosomes. After secretion, these tetramers assemble to form an extended microfibrillar network. The presence of alternative long α chains that may replace the α3 chain adds to the complexity of collagen VI assemblies. The lack of structural information hinders the in-depth understanding of the assembly process and of the impact of mutations that lead to muscular dystrophies.

The lack of expression of the novel long collagen VI chains in collagen VI α1 chain-deficient mice was a first indication for their participation in the assembly of collagen VI molecules (11). Further evidence supporting that the novel long chains may replace the α3 chain in assembly came from SAXS measurements and single molecule EM of their large N-terminal globular regions. The SAXS models of the novel long chains show monomeric structures that have a similar shape as that of the α3 chain N-terminal region. The SAXS results were confirmed by single particle EM of the N-terminal globular region of the α4 chain. The determined nanostructure as well as the *ab initio* SAXS model and the rigid body model all resemble the compact C-shape found for this region of the α3 chain. The consistently monomeric state indicates that self-interactions between the N-terminal regions are not involved at any stage of assembly.

**FIGURE 11.**
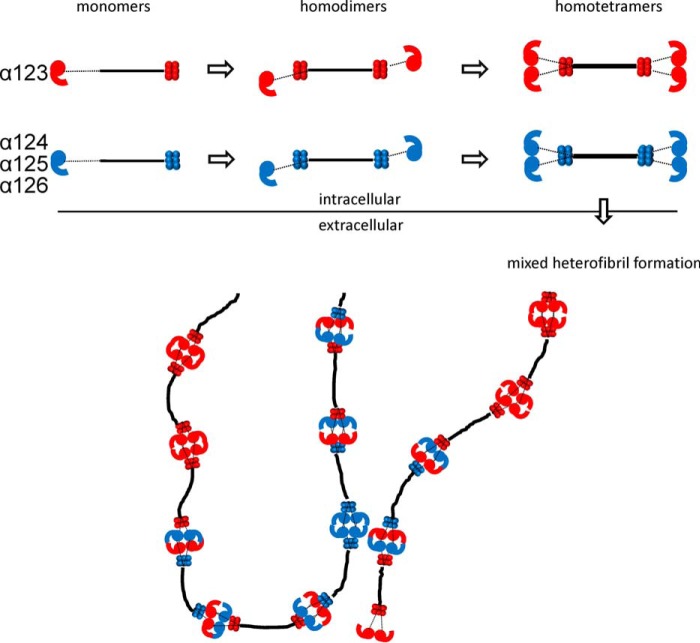
**Assembly of heterogeneous collagen VI microfibrils.** Schematic drawing of the assembly of the heterogeneous microfibrils. The composition of the schematically drawn microfibrils below reflects the composition of the mixed microfibrils containing the α3 and the α6 chain shown in Fig. 8. Collagenous domain, *black*; non-collagenous regions of the α3 chain, *red*; non-collagenous regions of the α4, α5, and α6 chains, *blue*.

Because of the striking similarity of the shape of the long chain N-terminal regions, we further investigated how these are integrated into microfibrils. Collagen VI purified from newborn mouse carcasses was studied by immunoblot analysis after composite SDS-agarose/PAGE. Under non-reducing conditions, collagen VI oligomers containing the α4 and α6 chains migrated close to oligomers containing the α3 chain, indicating that the novel long chains also assemble into homotetramers. However, as some bands co-migrate it could not be determined whether these are homotetramers containing only one long chain or heterotetramers. Interestingly, the α5 chain was detected only under reducing conditions, indicating that this α5 chain is disulfide-linked to other polypeptides or that α5 chain-containing tetramers are scarce and below the detection limit. However, antibody labeling followed by electron microscopy more clearly revealed how collagen VI containing the novel long chains assembles. When microfibrils from E14.5 mouse lung were labeled with affinity-purified, gold-labeled antibodies against the long chains, all four were shown to be part of collagen VI microfibrils with the typical beads-on-a-string appearance. These experiments unequivocally demonstrated that the assembly of collagen VI molecules containing the novel long chains must be very similar to those containing the α3 chain. Moreover, we could only detect homotetramers containing only one of the long chains α3, α4, α5, or α6 when studying individual tetramers or homotetramers as building units in microfibrils. In the microfibrils, homotetramers that contain either the same or a different long chain assemble end to end but with an overlap. The formation of mixed microfibrils of differing composition (schematically shown in Fig. 11) adds another level of complexity to the assembly of collagen VI. As the novel long chains have a restricted tissue distribution, it is likely that the mixed fibrils have specific functions in regions where a particular novel chain is expressed. One such function could be their integration into the extracellular matrix. As it was suggested that the N-terminal globule of the α3 chain projects out from the rest of the molecule, potentially providing interaction surfaces for other cell surface or matrix molecules (24), the different long chains could interact with alternative molecules. Indeed, by electron microscopy of skeletal muscle using gold labeled antibodies, it was shown that the α6 chain is restricted to the reticular lamina of muscle fibers but is absent at both the interface with the lamina densa and the basement membrane of capillaries (13). In contrast, the α3 chain is present at all these sites. The novel long chains may also alter the mechanical properties of the microfibrils, particularly as they contain significantly more cysteine residues than the α3 chain (Fig. 1). These could alter the microfibril flexibility by formation of additional disulfide bonds.

In contrast to the large N-terminal globular regions, SAXS models of some C-terminal globular regions indicated their involvement in assembly, as the C-terminal globular regions of the collagen VI α1, α2, and α4 chains, but not that of the α6 chain, appeared as dimers. Indeed, according to the most recent assembly model (Beecher *et al.* (24)), tetramer formation, but not dimer formation, may require a contact between α1 and α2 chains, and therefore self-interactions could play a role in tetramer formation. This is supported by the presence of dimers but the absence of tetramers in a patient with autosomal recessive myosclerosis myopathy lacking the C2 domain of the α2 chain (17). Moreover, *in vitro* transfection experiments indicated a role for the C2 domains of the α1 and α2 chains in collagen VI assembly (48).

The analysis of the structure of the non-collagenous regions of collagen VI gave further insight into their role during assembly and for the composition of microfibrils. The presence of three novel long chains adds to the complexity of collagen VI microfibrillar assemblies. Further experiments are needed to analyze differences between collagen VI microfibrils of different composition, *e.g.* their integration into the extracellular matrix, their exact localization, and their contribution to the mechanical properties of the tissue.

## Author Contributions

C. B., M. P., and R. W. conceived and designed the experiments. C. B., C. P. B., M. M., S. L., and T. M. performed the experiments. C. B., C. P. B., M. M., M. P., R. W., and T. M. analyzed the data. P. B. contributed essential materials. C. B., M. P., and R. W. contributed to the writing of the manuscript. All authors reviewed the results and approved the final version of the manuscript.

## Footnotes

5 Homotetramers are tetramers of monomeric heterotrimeric collagen VI molecules composed of one α1 chain, one α2 chain, and one of the alternative α3, α4, α5, or α6 chains see Fig. 11.

## Footnotes

4 The abbreviations used are:

VWA: von Willebrand factor type A domain
SAXS: small-angle x-ray scattering
EOM: ensemble optimization method.
